# Reforming regulatory relationships: The impact of medical revalidation on doctors, employers, and the General Medical Council in the United Kingdom

**DOI:** 10.1111/rego.12237

**Published:** 2019-01-09

**Authors:** Abigail Tazzyman, Marie Bryce, Jane Ferguson, Kieran Walshe, Alan Boyd, Tristan Price, John Tredinnick‐Rowe

**Affiliations:** ^1^ Alliance Manchester Business School, University of Manchester Manchester UK; ^2^ Plymouth University Plymouth UK; ^3^ Faculty of Medicine and Dentistry Plymouth University Plymouth UK; ^4^ Faculty of Health & Human Sciences Plymouth University Plymouth UK

**Keywords:** accountability, health services, medicine, professionalism, regulatory governance

## Abstract

In 2012, medical regulation in the United Kingdom was fundamentally changed by the introduction of revalidation – a process by which all licensed doctors are required to regularly demonstrate that they are up to date and fit to practice in their chosen field and are able to provide a good level of care. This paper examines the implications of revalidation on the structure, governance, and performance management of the medical profession, as well as how it has changed the relationships between the regulator, employer organizations, and the profession. We conducted semi‐structured interviews with clinical and non‐clinical staff from a range of healthcare organizations. Our research suggests that organizations have become intermediaries in the relationship between the General Medical Council and doctors, enacting regulatory processes on its behalf and extending regulatory surveillance and oversight at local level. Doctors’ autonomy has been reduced as they have become more accountable to and reliant on the organizations that employ them.

## 1 Introduction

The organization and management of medical work has long attracted considerable attention among academics and healthcare policymakers alike. Much of this work has focused around the concept of professionalism and autonomy (Kuhlmann & Burau 2008; Rees 2008; Exworthy 2015). Research specifically considering the regulation of medical work is less common. Such research has focused on how changes to regulation have been responded to by the medical profession, and what this means for the profession's relationship with the regulator, government, and wider society (Trubek *et al*. 2008; Waring *et al*. 2010; Chamberlain 2014; Archer *et al*. 2017).

Medical regulation in the United Kingdom (UK) was fundamentally changed in 2012 with the introduction of revalidation – a continued competency process by which “all licensed doctors are required to demonstrate on a regular basis that they are up to date and fit to practice in their chosen field and able to provide a good level of care” (General Medical Council [GMC] 2013). Before revalidation was introduced, once doctors qualified and joined the medical register they were only subject to regulatory scrutiny if concerns were raised with the regulator by patients, colleagues, employers, or others about their performance and fitness to practice. The introduction of medical revalidation followed more than a decade of protracted debate between the regulator, government, the Department of Health, and the profession concerning the regulation of the profession and appropriate methods for assuring the quality and safety of medical practice (Irvine 2003; Archer *et al*. 2015). In common with other such “continued competency” systems internationally (Horsley *et al*. 2016), revalidation has brought an extension of regulatory oversight of doctors’ practice throughout their post‐qualification careers. To date, most published work on revalidation has focused on the views and experiences of doctors engaged in the process (UMbRELLA 2016), often explored within particular specialty groups (Dale *et al*. 2016; Royal College of General Practitioners 2016; Stern 2016; Williams *et al*. 2016; Morris & Withnall 2017), on its impacts on intra‐professional relations (Chamberlain 2012), and on the profession's relationship with the medical professional regulator, the General Medical Council (GMC). However, focusing exclusively on the professional impacts of the introduction and implementation of revalidation neglects an important dimension of the policy, which sets it apart from past medical regulatory schemes. For the first time, revalidation has brought professional regulatory activity and oversight formally into the organizational sphere, placing statutory responsibility on organizations for the doctors they employ, and providing considerable legal powers and duties (GMC 2013). In this paper we consider how the implementation of revalidation has changed relationships between medical professionals, the organizations they work in and the statutory professional regulatory body. We begin by providing some background information on recent regulatory change in the UK medical profession, including revalidation, and set these in the context of relevant theoretical literature. We then detail our methodological approach, set out our findings, and discuss their implications for the relationships between professional and organizational regulation.

### 1.1 Background

Revalidation has been implemented against a complex backdrop of existing relationships: those between doctors as professionals and the institutions within which they undertake their work; and between doctors as professionals and their professional regulator; as well as the historically less well developed relationship between healthcare organizations and the professional regulator. It can be seen as part of an ongoing move of governance “upstream” (away from the regulator), with the aim of producing earlier, local resolution for performance concerns and more active and ongoing local oversight of performance. The purposes of revalidation have been much contested, with some regarding it as an important mechanism to protect patients and improve the quality of care and others regarding it as an unwelcome and bureaucratic system to exert greater control over doctors with few real benefits (Archer *et al*. 2015; Tazzyman *et al*. 2017). This move brings the work of regulation into the organizational sphere, a situation in which complex relational and governance issues already exist. In order to locate our analysis within these varied dynamics, it is important to first consider how these relationships have developed to date.

The GMC traces its history back to the 1858 Medical Act (21 & 22 Vict c 90). Importantly, this direct relationship between individual professionals and a professional regulatory body was established long before the creation of the National Health Service (NHS) in the UK, and was founded on a model of medicine that saw doctors practising autonomously – both in general practice and in hospital medicine. Historically, such autonomy was seen as a defining characteristic of professional work (Durkheim 1957; Freidson 2001).

However, during the 20th century, as health care provision expanded in scope and became increasingly complex/specialist, doctors were brought into ever closer employment relationships with provider organizations. In the UK, state provided health care in the form of the NHS particularly exemplified this shift, although independent provision continued alongside the new state service. Despite such changes, the management of poor performance was still not usually dealt with through formal professional regulatory or disciplinary mechanisms but instead locally and informally, meaning that doctors still retained considerable autonomy (Rosenthal 1995; Salter 2001; Chamberlain 2009).

More recently, doctors’ influence within healthcare management and autonomy within their own practice has been seen by many as having declined as a result of a wider societal shift from trusting professions and institutions to holding them to account (Power 1997). Governance was brought increasingly into the organizational sphere. Changes included an extension of clinical governance processes driven by central government, with healthcare organizations required to monitor clinical performance, and an increased role for non‐medical managers in health care (Wright 2009; Waring *et al*. 2010). There was also an increased focus on transparency and accountability of health care, including medical practice. In addition, reforms to public services involving marketization, competition, and increased internal and external accountability, often termed New Public Management, are argued to have contributed to this trend (Waring *et al*. 2010).

Regulatory arrangements for medical practice remained, however, largely unchanged, with the GMC responsible for professional regulation, assuring standards of medical education, and regulating post‐qualification doctors’ practice and behaviour through its Fitness to Practice procedures in instances of alleged wrong‐doing (Chamberlain 2014). Beyond this, doctors were expected to be either self‐regulating – practising in line with a shared understanding of professional standards, resolving any transgressions within the profession and often informally – or were subject to the “three wise [men] procedure,” in which senior doctors would act as an investigating committee (Rosenthal 1995). The regulation of doctors in this manner was increasingly challenged, and a contentious and, at times, confrontational debate about medical regulatory reform ensued (Irvine 2006; Saks 2014; Adams 2017). A series of high profile medical scandals and resulting public inquiry reports, most notably the Shipman and Bristol cases, called for change and criticized the GMC for prioritizing the protection of doctors over patient safety, and for failings in the way in which fitness to practice cases were dealt with (Kennedy 2001; Smith 2005). In response to the Bristol inquiry report (Smith 2005), the government created a new statutory body, the Council for Healthcare Regulatory Excellence, to oversee the performance of all health profession regulators, including the GMC, giving it legal powers to challenge fitness to practice decisions it deemed unduly lenient in court and to audit fitness to practice processes (Professional Standards Authority 2016). The governance of the GMC was reformed, reducing its council in size and making both professional and lay members appointed (rather than elected by the membership). New arrangements for independent adjudication and other changes to fitness to practice proceedings were also introduced (Donaldson 2006; Department of Health 2007). Importantly, the legislation enabled the introduction of medical revalidation, the last but biggest piece in the jigsaw of regulatory change.

Revalidation thus forms part of the wider reforms of the regulation of the medical profession (Hasselbalch 2015). The implementation of revalidation was the subject of long negotiations between the GMC, Department of Health, and professional bodies (Archer *et al*. 2015). Revalidation fundamentally changed how doctors were regulated by making the regulator–doctor relationship a continuing one throughout a doctor's medical career and bringing all practising doctors into that relationship, not only those the subject of complaints or concerns. Moreover, revalidation brought professional regulation into the organizational sphere, moving the governance of doctors upstream to be dealt with earlier and more locally in healthcare organizations. As such, revalidation has decisively engaged healthcare organizations in regulatory activities in a new way and in turn sought to reduce the volume of governance issues that are brought to the GMC. Revalidation, in repositioning where regulatory practices occur, means that such activities now take place in contexts in which complex and varied managerial and governance issues and systems exist.

The boundary between the medical profession and the organizations in which they are employed is not straightforward, and doctors both individually and collectively can be construed as acting on behalf of their profession, their organization, or both. An increasing stratification of the profession has been noted (Freidson 2001; Waring 2014), with the emergence of professional elites who exercise or enact governance on behalf of the profession, the regulator, and of organizations or institutions. Arguably, revalidation reinforces such stratification and extends the reach and intensity of organizational oversight (Bryce *et al*. 2018). Previous research on regulation and the medical profession has extensively theorized ongoing reforms, giving particular focus to what they mean for the relationship between the profession and the regulator and/or state. Reduction in self‐regulation and autonomy, for example, has been understood by some as a form of deprofessionalization (Schlesinger 2002; Bezes *et al*. 2012). This interpretation, however, has faced criticism for oversimplifying change, focusing solely on what the profession was seen to lose, and positioning those within it as both passive agents and in opposition to all managerialism (Harrison & Ahmad 2000). In contrast, restratification theory (Freidson 1985, 1994, 2001) situates the profession as active in response to ongoing changes (Kirkpatrick *et al*. 2005). From a restratification perspective, the profession responds to increased management by restructuring itself in order to maintain autonomy at a collective level, establishing new elite groups to deliver oversight and act as mediators between the profession and the state and regulator. In the development of this argument, a need to critique the extent to which professions are understood to be able to restratify was raised (Coburn *et al*. 1997; Waring 2014). Consideration of how much control the profession has over not only the content but also the context of medicine and whether organizations (such as the GMC) are co‐opted for governmental agendas was argued for.

The Foucauldian concept of governmentality (Foucault 1977, 1991) has been utilized to help understand such changes to regulation and governance of the medical profession (Waring 2007, 2014; Chamberlain 2012). Governmentality proposes that state regulation does not necessarily bring about conformity through explicit direction and enforcement, but rather through normalization and categorization (Foucault 1977, 1991). By enacting the requirements and expectations of external regulation (such as appraisal or collection of supporting information about performance in the case of revalidation), internalization can occur. As a result of this internalized self‐regulation, governance from state level is achieved with the reduced need for external oversight (Exworthy 2015). Analysis from a governmentality perspective enables the exploration of the contours of power within reforms, which is particularly useful when looking at the renegotiation of professional space, as in this paper (Doolin 2002). From this perspective, power is conceptualized as a system of relationships woven throughout society and it is within the “place” of the individual that power is enacted. Power is thus understood as a relation or interaction in which people are positioned (Foucault 1977), rather than something possessed. Understanding power as something constantly under negotiation allows not only for changes in the location of power to be identified but also for the ongoing relational work of power negotiations to be explored and existing explanations of the profession's response to regulation change to be considered. Governmentality thus enables an understanding of the significance of changing accountabilities between those involved in the regulatory relationship and the exploration of these changes as power negotiations.

### 1.2 How revalidation works

Revalidation is carried out in a five yearly cycle, informed by doctors’ participation in annual appraisals. For most doctors, appraisals are organized and conducted within an organizational structure, with the appraiser and appraisal meeting being supplied by the organization with whom the doctor is contracted (in the case of primary care physicians), or by which they are employed (for those in secondary care). This, along with the requirement that doctors collect supporting information about their practice to be reviewed at appraisal, which is often collected by the organization and supplied to the doctor for their appraisal, gives healthcare organizations an important role within revalidation. Indeed, all organizations employing or contracting doctors (termed “designated bodies” in the language of the legislation) are required by legislation to appoint a senior doctor to act as Responsible Officer (RO) who must ensure that the organization provides a suitable appraisal process, and who is charged with monitoring the fitness to practice of all doctors within their organization alongside wider clinical governance responsibilities (Medical Profession (Responsible Officers) Regulations 2010; GMC 2018). The RO role is a hybrid position; ROs are themselves members of the medical profession and a manager of other doctors, in a position that combines responsibility to the employing organization and to the professional regulator (Bryce *et al*. 2018). These responsibilities are summarized in Table 1.

**Table 1:** Responsibilities for revalidation

Doctor	Organization/ Designated Body	GMC
Licensed doctors who are not trainees must: Have a connection to one organization (known as a designated body), a Suitable Person or carry out an annual return directly to the GMC Take part in regular appraisal Collect supporting information for appraisal: 6 types CPD Significant events Review of complaints and complements Quality improvement activity Feedback from colleagues Feedback from patients Reflect on supporting information	Organizations are required by the GMC to provide: An RO – new role, responsible for the revalidation recommendation An up‐to‐date appraisal system and ensure every licensed doctor has a regular appraisal A sufficient number of trained appraisers Clinical governance systems that can provide supporting information Policies and systems for identifying and responding to concerns about doctors Link with other organizations where doctors work, so information about their practice can be shared RO makes one of three possible revalidation recommendations to the GMC: Revalidation Deferral (request for GMC to provide more time for revalidation decision); does not affect licence to practice Non‐engagement (can lose licence)	As the regulator, the GMC is responsible for: Setting guidelines Making the final revalidation decision based on RO's recommendation (to revalidate; how much time to provide for deferral and whether to refer to a Fitness to Practice Panel) Provide an Employer Liaison Service to help ROs with revalidation and the management of concerns about doctors

CPD, Continued Professional Development; GMC, General Medical Council; RO, Responsible Officer.

Revalidation, then, places requirements not only on individual doctors, but also on the organizations within which they work. The regulation and governance of organizations is not within the GMC's statutory remit, but, as many of revalidation's component mechanisms draw on organizational data systems and processes, such as appraisal, the introduction of this professional regulatory intervention has required actions and resources at organizational level. Organizations with designated body status have been given new responsibilities and obligations to their doctors’ as a result of the non‐negotiable requirements of revalidation (Table 1). In doing so, it divides responsibility for overseeing medical performance between the medical regulator and organizations. While the core relationship focused on by revalidation is that between an individual doctor and their professional regulator, the tripartite distribution of responsibilities inevitably brings organizations into the dynamic. Healthcare organizations – especially those within the NHS – can be seen as part of the state apparatus and acting as a conduit of state power and policy. They do, however, have some degree of agency as to how to apply that power and meet their obligations to the state (Hoque *et al*. 2004). Exploring the role played by organizations in shaping the implementation of revalidation is therefore key to understanding this new regulatory landscape. Governmentality has been chosen as the theoretical perspective because it goes beyond examining the formal structures of the state to examine how the power of the state can be exerted through a range of organizations in the context of health care, and offers a fresh perspective on this topic.

This paper sets out to answer two key questions. Firstly, what are the implications of revalidation on the structure, governance, performance management, and oversight of the medical profession? Secondly, how has revalidation changed the triumvirate relationship between the regulator, employer organizations, and the medical profession? The Foucauldian concept of governmentality is used as a starting perspective to answer these questions (Foucault 1977, 1991).

## 2 Method

### 2.1 Sample

This paper draws on qualitative data collected as part of a wider study to investigate the implementation of medical revalidation and its impacts on and for healthcare organizations. Healthcare organizations were recruited following a national survey of ROs (Walshe *et al*. 2017). Using this information, 15 healthcare organizations were recruited in order to ensure coverage across types, settings, performance (appraisal rates, self‐assessment), and geographic regions. In each organization, procedures, board reports, and role descriptions relating to revalidation or the management of medical work more broadly were reviewed. We used this information to develop an initial understanding of the organization's structure and to inform a list of potential interviewees focusing on those in key roles relating to revalidation, managing medical work and doctor's performance, appraisal, and wider governance and quality and safety processes. In all, we conducted 80 interviews with 79 participants. Healthcare organization and participant details are listed in Table 2.

**Table 2:** Healthcare organizations and participant details

Healthcare organizations by type	Participant roles	
Licensing agency for locum doctors	Medical director Operations director Responsible officer	Locum x2 Appraiser
Locum agencies A, B, C, and D	Responsible officer x2 Clinical governance manager Medical director	Locum x6 Recruitment director
CIC regional community healthcare provider	Former responsible officer Head medical staffing† Responsible officer	Director of professional practice, safety and quality
Mental health charity	Appraiser x2 Senior HR business partner Responsible officer† Deputy director of quality and compliance† AMD	PA and admin† Revalidation support officer and HR business partner AMD for medical training, education, and medical regulation
NHS acute hospital and community health care foundation trust	Previous AMD AMD x2 (patient safety; revalidation)	Revalidation administrator Responsible officer and medical director
NHS acute hospital foundation trust (medium)	Responsible officer AMD and deputy responsible officer Head of clinical governance	Revalidation manager Head of clinical effectiveness
NHS mental health foundation trusts A and B	Chief nurse and executive director of operational clinical services Director of HR Responsible officer x2 Appraisal admin Deputy responsible officer	Medical director x2 Revalidation manager Appraisal lead Head of risk Complaints manager
NHS England area teams A and B (primary care)	Head of inspection – CQC Clinical lead primary care workforce development and education Appraisal admin x2 Senior appraiser x3 Responsible officer x2 Head of revalidation x2 Program manager Senior project officer Appraisal lead	Assistant director fitness to practice Practice manager Senior project officer for revalidation LMC secretary LMC CEO Deputy medical director GP appraisal and revalidation manager
Private healthcare provider	Responsible officer National director of clinical services Medical director	Consultant liaison and revalidation manager Clinical governance director
Small hospice charity	Responsible officer	

† Interviewed twice. AMD, Associate Medical Director; CEO, Chief Executive Officer; CIC, Community Integrated Care; CQC, Care Quality Commission; GP, general practitioner; HR, human resources; LMC, Local Medical Committee; NHS, National Health Service; PA, personal assistant.

### 2.2 Data collection

Seven researchers conducted interviews between 2015 and 2017. The research team developed an initial interview guide based on knowledge gained from reviewing organizations’ documents and literature on the management of medical performance. Having conducted initial analysis on the first batch of data, we developed a second interview guide to further explore emerging themes and to purposefully focus on filling gaps in the data to reach a point of apparent data saturation (Green & Thorogood 2013). Interviews were conducted either in person or by telephone, according to the preference of the interviewees, and digitally recorded and professionally transcribed for analysis.

Ethical approval for this study was awarded by the University of Manchester ethics committee (REC 15028).

### 2.3 Analysis

We imported transcribed data into Dedoose qualitative data analysis software (Dedoose 2013). The research team developed an initial coding framework through team discussion, trial, and revision based on our collective interpretation of the data. We used the Dedoose software to enable blind coding and verification of code application to check the consistency of analysis. All authors discussed coding and interpretations at regular intervals throughout the analysis phase of the study during collaborative meetings. As well as the established initial codes, we added further codes to the framework inductively as appropriate and then coded across all transcripts (Fereday & Muir‐Cochrane 2006).

We followed with an iterative process of analysis in which the coded data was reviewed further, specifically investigating the key themes identified across the dataset. The team determined chosen themes through discussion of the initial coding findings until a consensus was reached based upon the significance and prevalence of each topic. We extracted all data potentially relevant to organizational, professional, and regulatory relationships. Two researchers re‐read the extracted data until satisfied that all subthemes had been identified. These two researchers then refined the subthemes through a process of discussion, which was agreed upon by the wider research team. We focused our analysis on the analytical framework, moving between the data and the literature in order to refine and situate our findings (Braun & Clarke 2006, p. 84).

## 3 Findings

Our findings centre on three main themes: the organization as hub/core, expanded surveillance/ audit culture, and doctors as employees. The first of these themes (organization as hub/core) focuses on the centrality of organizations to the delivery and mediation of revalidation. The second theme (expanded surveillance/audit culture) explores the capability of organizations to oversee and control doctors. The final theme (doctors as employees) examines the impact of revalidation on the position of doctors in organizations and of the profession more broadly.

### 3.1 The organization as hub/core

The organizations that employ doctors and act as designated bodies are vital for the operationalization of revalidation. While revalidation as a policy is about a relationship between the regulator and its oversight of an individual doctor, in design and implementation it introduces organizations as intermediaries in that relationship in a new way. In practice and in legislation, revalidation is mediated through and dependent upon the organizations in which doctors work for its implementation.

The GMC is not an organizational regulator; however, the statutory responsibilities placed on organizations by the legislation associated with revalidation, necessitated many organizations to introduce new or adapt existing processes. This was especially true of processes for collecting and collating clinical governance information, appraisal processes, and wider safety and quality systems:

We've made quite a lot of changes. We've put in place a different leadership structure, so we've got myself, who supervises a number of Clinical Directors, who supervise a number of Clinical Leads, who supervise a number of Consultants.^1^

There is clearly a role of process that has to be followed, in other words, to make sure that we offer a means of supporting our doctors, to relicense with the GMC, we need to manage that system, and I do that on behalf of [RO name], but every doctor's sign off has to be discussed with [RO name]. So effectively I do all the work, and then we have a finalizing meeting. And then also, within that role, is the management of the appraisal system, so we need to obviously recruit and train appraisers, we need to maintain the training of appraisers, and then we need to conduct the appraisal system in a manner that is appropriate for all permanent staff.^2^

In this regard, professional regulatory intervention has driven change at organizational level with organizations situated as the mediators of revalidation. The significance of organizations as a hub for the delivery of revalidation and their position as core to its operationalization is particularly well illustrated by examples of the difficulties faced by those outside or on the edges of organizational boundaries. Doctors positioned outside of organizations, such as those working in private practice or locums, featured in participants’ narratives as the weak link in revalidation. They were almost always identified by others or themselves as unable to access the support provided by organizations, such as appraisal delivery, information collection, and continuing professional development, making the revalidation process a more difficult and onerous task:

It's getting the evidence. So it's difficult for a doctor who is transient, moving from place to place, to get patient feedback for instance. Or colleague feedback, because people say, well you've only worked here for two weeks, I don't really know you well enough to give you feedback. Also if a significant event arises about something, they may well not find out that it's happened [be]cause they may have already left or moved somewhere else.^3^

A locum out there is quite a risky … from a quality point of view a locum can work in one practice one day, one practice another, so it's quite hard to oversee their performance.^4^

This group of doctors were also described as those furthest outside “the net” of revalidation, which was frequently seen as an inappropriate or insufficient approach for the performance management of those in such working contexts. Although a GMC policy, without the support and operationalization of employing organizations, the robust form of regulation cannot encompass all doctors as intended.

Changes in the relationship and communication channels between the regulator and healthcare organizations further highlighted the new significance of organizations for regulator operationalization. Each healthcare organization has, as a point of contact with the GMC, an Employer Liaison Adviser (ELA), who is the designated first point of contact for organizations with the GMC, and from whom organizations can obtain advice and support. This new role is identified as having improved organizations’ ability to communicate effectively with the GMC and as an important source of informal guidance on revalidation:

We have a very good relationship with both of ours [ELAs]. We've got another one now, but we've always got on really well with them. [RO] runs lots of things by them informally. They're very good, they'll get back to you very quickly if they don't know something. They know the answer to most questions straightaway. We've found it really, really helpful in this trust … I appreciate those meetings.^5^

Acting as brokers of information, the ELAs are fundamental to the regulatory relationship between organizations and the GMC, symbolizing a change in this relationship and a recognition on the part of the GMC of the central role and significance of organizations for revalidation and the governance of doctors.

### 3.2 Expanded surveillance/audit culture

The changes revalidation brought to the relationship between organizations, doctors, and the regulator presented an opportunity for organizations to expand their surveillance of doctors’ performance and an increase in audit culture. Through compulsory appraisal, doctors are now more accountable to organizations, with non‐engagement now carrying the risk of losing licence to practice, unlike before. Revalidation is described as having improved the collection and triangulation of performance data, which in turn has reportedly contributed to organizations’ increased ability to monitor their doctors.

The responsibility of ROs to sign off doctors as fit to practice across their whole scope of practice led many organizations to invest in improving their performance and governance information collection and recording systems in order to provide the necessary oversight. The necessity of this oversight from the RO and the doctors’ need for performance information for their appraisal meant organizations had a new legitimacy and authority to collect such information where before this could have been challenged:

So we gather data on their involvement in any learning, from serious incidents, complaints, that sort of thing. We have a section called quality indicators, and in that we'd also put other stuff like if they make named requests a lot to pharmacy, so doctors who want to prescribe non‐formulary products, that sort of thing. Anything that we think is a benchmark of quality, that goes into their appraisal.^6^

Although on the whole, organizations collected the required information for appraisal on behalf of their doctors, it was still the responsibility of individual doctors in most cases to obtain this information from their organization and take this to appraisal. This meant that doctors were able to pick and choose what they presented and revealed in an appraisal. Instances of or the possibility of doctors gaming the system by not declaring negative information, or treating revalidation like a tick box exercise and merely jumping through the hoops, were raised. In part to combat the tick box approach, as well as to meet the requirements of RO oversight and the need to make an informed decision based on the whole scope of practice, organizations began to improve not only their data collection but also the triangulation of that data and the bringing together of different information sources to provide a more comprehensive overview of doctors practice rather than isolated information. Although full triangulation has not yet been delivered in all organizations, improvements have consistently been noted, as well as intentions to further improve the oversight organizations have over their doctors’ performance:

When a doctor has had an investigation, once that investigation is closed, we then write to them and say, you've been subject to an investigation, we would like you to reflect on that in your next appraisal. And then we use revalidation or appraisal as a way of auditing whether they are engaging with the process. So, we don't tell the appraiser, but if the information is not put on to the next year's appraisal, we would then refer them back to the GMC, or use the non‐engagement, that's how we make the doctors concerns really into revalidation.^7^

So before, you know, you might get a patient complaint about somebody, there might be a clinical concern dealt with there, something dealt with something. And it's bits of information all over the hospital but actually nobody pulling it together, you know, nobody thinking actually that was the doctor that did … whereas now, I think we're picking up on that much better … And that's been a big part of my role, I suppose, and why I now sit in HR is to pull all that sort of stuff together. And I think a lot of this stuff, the medic stuff, has always been dealt with by other medics and HR haven't always been involved in it, so it's not always been documented very well. It's much better documented now.^8^

The RO role was not the only emergent managerial function identified as having strengthened managerial oversight of doctors’ performance. For example, one participant identified the revalidation lead within their organization as having a key role in triangulating information about doctors and identifying potential concerns earlier than may previously have been the case:

I think the bit we didn't have which has helped considerably is having a revalidation lead, so someone who's actually got the time to look [into] and triangulate things that may have been a bit lower on the radar. So I think we are picking up things maybe slightly earlier than them causing, you know, being flagged up as a serious incident or whatever before that stage if there's been some sort of complaints or some lower level incident reporting.^9^

Another respondent, when referring to doctors’ work as clinical or educational supervisors for postgraduate specialty trainees, reported that systems for sharing information had improved, and this was helping to ensure that doctors holding these roles kept up to date with their responsibilities:

I work closely with the postgrad team, in relation to education and clinical supervisor. […] So we work pretty closely, we've got systems, and we're developing, they're developing an escalation policy, as they've called it, which links to our reminder letter system. So not only are they getting me mithering them, but they're gonna get the postgrad team mithering them as well. Because we've got a lot of clinical, especially supervisors, so we have to make sure they're in date, to keep up that role. So that's working, and that's, over the last 12 months. And the system, again, was tweaked, to allow them to use the system to demonstrate evidence for those roles, without having to do it separately. So that's made it easier for them, and then it's easier for us to monitor it, and the postgrad team have got access to the system for that section. So they can see when somebody's had an appraisal, but she knows that I'm chasing them, so she'll know where we're up to, and she'll go, do you know where this person is up to, and how much chasing have you done … So I can keep her informed, especially like, the ones that have been really naughty, so I can tell her to put them on her radar as well as my radar. So we work closely in that respect.^10^

Improved connections between performance systems and better processes for information sharing support organizations ability to better monitor doctors’ performance and to intervene when deemed necessary.

### 3.3 Doctors as employees

Changes in the relationship between organizations and professional regulation brought about by the implementation of revalidation have, as described above, shifted regulatory processes “upstream” into the organizational sphere. Having provided an opportunity for organizations to expand their surveillance of doctors’ performance through strengthened mechanisms of accountability and increased requirements to participate in processes of appraisal, for example, the implementation of revalidation has impacted on the position of doctors within the healthcare workforce.

From the perspective of those responsible for managing medical performance, the advent of revalidation has brought doctors more into line with other healthcare professionals by bringing them under increased scrutiny and reducing prior hierarchical differences in approaches to managing different professional groups:

It's probably given us greater clarity, because one of the things I did notice when I had to write that policy [for organizational revalidation systems] is that we had one about conduct for all staff, and it was very clear at the bottom it just said, this does not apply to medics. And, I think that was because everybody knew it was such a minefield.^11^

There could be a danger culturally. And it might be anecdotal and stereotypical what I'm going to say, but that doctors might be a bit precious and might be treated a bit differently than other groups. And the revalidation process leaves them a bit more accountable as other professions have to be accountable, in a way that culturally might not have been the case in the past. So doctors were always, you know, untouchable and that kind of thing, might have been one perception in the past.^12^

Revalidation then is seen at least by some involved in managing doctors’ work, as having countered or negated any lingering claims by doctors to be exempt from managerial oversight or to merit special treatment in or by organizations. Revalidation has provided organizations with more authority over doctors and in turn has increased doctors’ accountability to them. Many organizations have used this development to their own advantage. In this vein, revalidation is seen as offering a means by which doctors can be compelled to participate in organizational management processes, serving to draw them into a “governed system” in new ways:

One of the things I think has been really helpful is the being able to articulate to doctors that actually being engaged with your work is the duty and I think that's one of the things that is a lifeline actually. So, the sort of, I couldn't care less about the organization, I just care about my patient, trying to explain that that isn't engagement. I'm not going to meet my line manager because I'm [a] doctor, I'm not prepared to hear that. It is possible to say to people, actually that, you know, engagement is about actually being part of a governed system, and I think that does help. I don't think people like it but it is not optional.^13^

Prior differences in approaches to managing performance between professions were the result of historical assumptions that medical professionalism and individual self‐regulation would ensure that doctors conformed to high standards of care and behaviour:

…I think in general people still regard their professional duty as something they take quite seriously, and the whole principle about being a professional is about self‐regulation and making sure your standards are of the highest and making sure you do keep up to date and you do operate with integrity and honesty and all the things that make good medical practice. […] It was that presumed professionalism in a way that was the old way, so what we're now saying is we need a bit of evidence behind that before we can make presumptions.^14^

One RO suggested that the requirement that doctors seek colleague feedback as part of revalidation may be contributing to a reduction in the perceived sense of independence or even superiority from doctors, given they may now be subject to critique:

People's behaviour is definitely improving […] I think people become more humble, in a way, so that stubbornness, big‐headedness, that type of thing actually becomes less of an issue because people know that you can't just behave in a randomly whatever way assuming I'm the authoritarian person, because people will actually feel … you are working with these guys where you're going to ask them later on, tell me about me.^15^

While some framed the impact of revalidation as being to diminish medics’ status and autonomy, others suggested that the personal responsibility placed upon individual doctors to participate in the process and to collect the requisite supporting information means that significant scope for action, and therefore perhaps autonomy, remains with the doctors themselves:

It's kind of giving that responsibility back [to] the professionals. As part of their revalidation, they're now expected to demonstrate, you know, how they are meeting their relevant guidelines at the moment. And as part of that, they will look to what data's available locally in terms of their supervision or their ward‐based information. But as you say, they also will come to us as well to help include that.^16^

However, elsewhere it was clear that while responsibility for meeting the requirements of revalidation as a regulatory process remains with the doctor, the degree to which this responsibility is exercised independently is limited. Rather some organizations monitor doctors’ compliance with the requirements, leaving it as their responsibility to submit supporting information to appraisal but intervening if this is not done:

So if you've had a complaint for example, it's expected, we don't in this trust put that onto the form. I know that some trusts would … somebody would populate your, you know, ePortfolio with that. But we would expect the person to declare that, so if there was a chance that something, bit of information that we are holding – which is all high level information – and there's nothing on the persons appraisal about that, then I would go back to the person to say, look, I know you were involved in a serious incident last year; this must have had a high impact on you, why are we not seeing anything on your appraisal, for example.^17^

In this instance, doctors’ independence in selecting information to discuss in their appraisal is limited by oversight from senior managers. Doctors are required to submit any complaints they have received about their practice as part of their appraisal portfolio each year, so this example shows organizational management processes ensuring that doctors are compliant with the process. However, it also belies the notion that placing responsibility for collecting supporting information with doctors may somehow support or protect their autonomy within this process. Rather, doctors are made accountable for their compliance with the requirements of the system, with organizational follow‐up and intervention if they do not conform.

Managers described their strengthened ability to manage doctors positively. One gave examples of instances in which potential problems with doctors’ behavior had been identified and acted upon, and that using colleague feedback and appraisal had enabled them to support the doctor to amend his approach and ultimately remain with the organization. Importantly, one managerial approach in this case had been to notify the doctor that information about the concerns with their performance would be passed onto new employers should he leave the organization:

We had a fairly newly appointed consultant … whose multi‐professional team was saying that they found him bossy and overbearing and not good at listening […] He wouldn't hear it, wouldn't hear it, wouldn't hear it. He was quite argumentative with his line manager but it was then … when his appraisal came round it was possible to have a discussion with him that actually he hadn't been reflective at all in his appraisal about almost anything. […] it was possible to meet with him and just say, look, you know, we need to get on top of this before you … or if you're going to move we would … your transfer form, we would have to say that we're in the midst of trying to help with this. So that, I think, helped him steady up and we were able to say that we would give him positive support, whether he stayed or whether he went. We … put a plan in place describing much more clearly the desired and undesired behaviors and he stayed.^18^

For many in managerial positions, therefore, the advent of revalidation is seen has having provided impetus for improved information sharing, extended management processes, and, overall, has increased oversight of the medical profession working within an organization. Moreover, such increased oversight has contributed to an apparent further degradation of doctors’ professional autonomy, tying them more closely to organizations’ managerial processes, as the regulatory requirements placed upon doctors by revalidation are fulfilled through systems largely operating within and mediated by those organizations.

## 4 Discussion

In this paper we have explored how the implementation of revalidation has impacted on the regulatory relationships between the GMC, healthcare organizations, and doctors. Revalidation is but one of a number of reforms, and its implementation has taken place against the wider backdrop of ongoing changes and parallel reforms that have impacted on medical autonomy in different ways (Scott *et al*. 2000; McGivern & Fischer 2010; Adams 2017). The reduction in medical autonomy attributed to these changes has been argued as necessary and timely (Chamberlain 2014; Saks 2014), although some would argue that it represents an unwarranted expansion of regulatory control (Williams *et al*. 2014). As a component of regulation, revalidation is an important innovation when contrasted both with systems of medical regulation internationally and with the regulation of other health professions in the UK (Spendlove 2013; Archer *et al*. 2017). Instead of doctors interacting directly (and relatively rarely) with the professional regulator, revalidation requires that appraisal and other information about performance instead be collected and considered at the organizational level, under the auspices of the RO.

The three themes from our findings, the organization as hub/core, expanded surveillance/audit culture, and doctors as employees, are interdependent. The working of each theme is dependent on the others, and it is as a result of the combination of all three areas that revalidation has shifted the regulatory process upstream and into the organizational sphere. Through revalidation, the GMC has delegated some responsibility for overseeing performance to organizations via ROs and in doing so has passed on most of the responsibility and much of the cost. Healthcare organizations are being made accountable for overseeing doctors in a new way, which has extended regulatory surveillance and oversight at a local level. For organizations, the implications of this upstream shift have centred predominantly on their increasingly important intermediary role in the relationship between the GMC and doctors. In operationalizing revalidation, organizations have to adhere to requirements set out by the GMC and through legislation, meaning that in many ways they themselves have experienced a form of regulation. Meeting these requirements frequently meant substantial changes to organizations’ governance practices and costly investment in infrastructure. While making organizations more answerable to the GMC and providing a new workload, the expanded surveillance of performance and the strengthened mechanisms of accountability brought by revalidation have provided them with new authority and leverage over doctors. Organizations are able to use revalidation to bring doctors more into the organizational sphere and to bring about adherence to their own agendas. Changes to the relationships organizations had with both doctors and the regulator fit well with the governmentality thesis, although at an organizational rather than a professional level in this instance. In enacting the GMC's guidelines, organizations self‐regulate but in turn use this to their own advantage to increase control of their doctors. The expectations of the GMC for doctors have been taken on by healthcare organizations who accepted the required self‐adaption to ensure these could be met.

As a consequence of the increased governance occurring at an organizational level, the position of doctors within the healthcare workforce has also been impacted. The need for doctors to revalidate and their reliance on organizations to facilitate that process has thus enabled organizations to legitimately increase their oversight of doctors and bring them into line with organizational agendas and priorities. The increased reliance of doctors on organizations for support to enact the revalidation policy is well evidenced by the difficulties experienced by those working outside organizational boundaries, doctors who are not employees, or who have a relatively transient or distant relationship to their employing organization. Because of the lack of organizational support available to such individuals, the regulatory relationship became increasingly problematic. The implementation of revalidation has highlighted shortcomings in the governance of this category of doctors, which remains unresolved. However, such concerns about the effectiveness of regulation for doctors outside organizational boundaries have been recognized by policymakers (Pearson 2017). Further changes to this area of regulation are likely as a result, highlighting the need for further research.

In addition to tying doctors more closely into organizations’ managerial processes, revalidation has also reduced prior hierarchical differences in approaches to managing different professional groups, positioning doctors as more like other employees. The new accountability doctors experience was framed, in particular, by organizations, the GMC, and doctors in roles contributing to the implementation of revalidation, as part of their professional obligations. This accountability has been positioned as existing alongside and perhaps in contrast to an apparent degradation of doctors’ professional autonomy and power. Although to some degree the RO role can be seen as a form of restratification overall, there has been some loss of professional autonomy and hierarchical power, both collectively and individually, as a result of revalidation (Bryce *et al*. 2018). Existing research has begun to consider possible hybrid roles and creative compliance, both more generally and as developed as a result of revalidation (Noordegraaf 2016; Bryce *et al*. 2018; Spendlove 2018). This work has identified an increase in hybrid roles within the professions (including medicine) and as a result professional work has become more “linked to outside worlds, especially organizational contexts,” thus becoming more accountable to them (Noordegraaf 2016, p. 785; Evetts 2011). What professionalism means is understood as an increasingly unstable category that is less isolated than before and requires more active legitimization. The findings of this paper echo this. Meeting the requirements of revalidation and being a more integral part of the organization has been positioned as a part of doctors’ roles and as central to a new more modern conceptualization of professionalism, both by those of the profession and those managing doctors. In the case of revalidation, the regulatory elements of the triumvirate relationships under investigation add a new driver in the development of what constitutes professionalism. Further investigation in these areas would be beneficial in light of the ongoing changes to medical autonomy, regulation, and the understanding of professionalism identified in this paper.

The findings of the research have some implications for our understanding of professional regulation internationally. Existing research looking at the different systems of continued competency for doctors has found that effectiveness is dependent upon the degree to which these systems are accepted and engaged with by the profession (Sehlbach *et al*. 2018a,b). This study has shown that the way in which doctors understand and conceptualize professionalism is important for how they accept changes to their accountability and regulation. Given the international nature of the medical workforce, and varied cultural conceptualizations of professionalism, considering the acceptance and success of such regulatory changes across and for those crossing national borders also warrants further research.

## 5 Conclusions

Revalidation was introduced with the aim of improving patient safety and the quality of medical care, however, it is too early to be able to determine what impact revalidation has had on safety and quality. Overall, the discourse used to articulate and enact the changes involved in implementing revalidation is primarily one of accountability, essentially concerned with to whom doctors, as a profession, should answer and to what degree.

Accountability is in many ways the key point of convergence to determine the impact of revalidation's on the triumvirate relationship investigated. Changes to each party's domain of accountability has altered the way these three stakeholders – the regulator, the medical profession, and employer organizations – relate to and interact with each other and the way in which the medical profession is governed. Organizations have become intermediaries in the relationship between the GMC and doctors, enacting regulatory processes on its behalf and extending regulatory surveillance and oversight at local level. As the importance of organizational ties strengthened, doctor's autonomy was reduced and the extent of organizational oversight increased.

What it means to be a doctor has been reframed through revalidation in ways that strengthen organizational accountabilities and make the organizational setting central to systems of oversight, changes compatible with ideas of governmentality.

## Notes

1 Interview 21 – RO.

2 Interview 37 – Associate MD for Revalidation.

3 Interview 73 – LMC CEO.

4 Interview 59 – Senior Project Officer.

5 Interview 52 – Reval Manager.

6 Interview 52 – Reval Manager.

7 Interview 76 – RO.

8 Interview 52 – Reval Manager.

9 Interview 40 – AMD and Revalidation Lead.

10 Interview 44 – Reval Manager.

11 Interview 84 – RO.

12 Interview 22 – Director of Professional Practice, Safety and Quality.

13 Interview 31 – RO.

14 Interview 41 – RO.

15 Interview 54 – RO.

16 Interview 56 – Complaints Manager.

17 Interview 35 – Previous Associate MD.

18 Interview 31 – RO.
